# Lymph Node Dissection During Radical Nephro-Ureterectomy for Upper Tract Urothelial Carcinoma: A Review

**DOI:** 10.3389/fsurg.2022.852969

**Published:** 2022-03-24

**Authors:** Arthur Peyrottes, Gianluigi Califano, Idir Ouzaïd, Paul Lainé-Caroff, Thibaut Long Depaquit, Jean-François Hermieu, Evanguelos Xylinas

**Affiliations:** ^1^Department of Urology, Bichat-Claude Bernard Hospital, Assistance-Publique Hôpitaux de Paris, Paris University, Paris, France; ^2^Urology Unit, Department of Neurosciences, Reproductive Sciences and Odontostomatology, Federico II University of Naples, Naples, Italy; ^3^Urology Unit, Military Teaching Hospital Sainte Anne, French Military Health Service, Toulon, France

**Keywords:** lymph node dissection (LND), upper tract urothelial carcinoma (UTUC), nephroureterectomy, anatomical templates, review

## Abstract

Although lymphonodal dissection is well-accepted for muscle-invasive bladder cancer management, its role is still debated during radical nephroureterectomy (RNU) for upper tract urothelial carcinoma (UTUC). The aim of this study was to summarize the current knowledge concerning the indication, anatomical template, prognostic, and therapeutic roles of lymph node dissection (LND) performed at the time of RNU. Quality control markers, such as the number of lymph nodes (LN) removed, lymph node density, and safety of the different surgical approaches, were assessed. We performed a narrative review using the PubMed and ClinicalTrials.gov databases. We identified and analyzed articles based on the practice and the role of lymph node dissection for non-metastatic UTUC. There are no clear guidelines regarding the indication of LND for UTUC, but aggressive tumors may beneficiate from lymphadenectomy since lymph node invasion is a clear independent poor prognostic factor, allowing for adjuvant treatments. It seems that an extended lymphadenectomy may provide therapeutic advantages as a higher number of nodes removed may be related to the removal of undetected LNs micrometastases and a subsequent improvement in recurrence rate and cancer-specific survival. Clear anatomical templates are thus needed based on the location and the laterality of the primary tumor.

## Introduction

Upper tract urothelial carcinomas (UTUCs) define urothelial carcinomas (UCs) with pyelocaliceal and ureteral localization (1). UTUCs differ from urothelial bladder carcinomas (UBCs) in practical, anatomical, and molecular aspects (2, 3). Localization in the upper urinary tract of UCs is relatively uncommon and is associated with aggressive behavior (4). Approximately, two-thirds of patients who present with UTUCs show invasive disease at the time of diagnosis (1, 5).

Individual risk stratification is the cornerstone for clinical decision-making of patients with UTUCs (4, 6–8). Tumor stage and grade are the main tumor-related prognostic factors (1). The presence of lymph node metastases represents an independent predictor of lower survival rates (9). The risk of nodal invasion increases with advancing tumor stage (10).

Radical nephroureterectomy (RNU) is the standard treatment of high-risk UTUC (1). Lymph node dissection (LND) performed at the time of RNU allows for optimal tumor staging, although its curative role remains controversial (1). Retrospective evidence still supports the LND procedure for all patients who are scheduled for RNU (1).

The management of UTUCs is significantly improving, thanks to considerable research efforts in the field. The advent of immunotherapy and multimodal strategy options pushes us to reevaluate the path of care we offer to patients. LND still remains an unmet need. The gray areas of knowledge mostly concern the therapeutic and prognostic role played by the LND at the time of the RNU.

The current review aimed to address this gap by shedding light on the updates and evidence studying the role of LND in the management of patients with UTUC.

## Materials and Methods

We performed a comprehensive review of the literature for current data on lymph node dissection performed during RNU for high-risk UTUC using the PubMed and ClinicalTrials.gov databases. The following terms were used: “upper tract urothelial carcinoma” OR “nephroureterectomy,” AND “lymph node dissection” OR “lymph node excision” OR “lymphadenectomy.” The literature search was limited to English-language and French-language articles. Articles were selected if they provided information on the indication, survival data, surgical technique, or safety of LND. Studies were excluded if they involved bladder cancer or used another surgical technique other than RNU. Other exclusion criteria were a lack of proven diagnosis, low-risk UTUC, and the absence of key information, such as hazard ratios, confidence intervals, and *p*-values. We performed our literature search covering the period from January 1, 2000 to September 1, 2021. Screening of titles and abstracts was done by the first author (PEYROTTES A.). Full-text quality and relevance were assessed by three independent authors (AP, GC, and EX). All authors participated in full-text sharing and determined the final list of publications that would be included.

### Indication of LND

Till date, there are no existing guidelines concerning the indication of LND and its performance during RNU. According to the EAU panel, “a template-based LND should be offered to all patients who are planned for RNU” (1). Many anatomical templates for lymph node dissection have been proposed and described. The standardization of LND indication and surgical technique is difficult due to the multicenter and retrospective design of the studies reviewed. In most of them, the indication to perform LND was at the surgeon's discretion based on clinical presentation, location, and laterality of the primary tumor.

Regional lymph nodes represent the most common metastatic site in UTUC (11) with an overall 25% incidence of node involvement, depending on tumor stage and grade (12). A retrospective study led by Kondo et al. (13) showed that the incidence of lymph node (LN) invasion was 5% for T2 tumors, 24% for T3, and 84% for T4. In patients with Tis/Ta/T, there were no positive LNs. LN invasion was present in 0%, 11%, and 35% of patients with G1, G2, and G3 tumors, respectively. Similarly, Roscigno et al. found that the proportion of pT1, pT2, and pT3-4 among pN+ was, respectively 5, 15, and 80%, and that 93% of patients with LN metastases had high-grade tumors (14). Other factors associated with nodal involvement are tumor necrosis, the presence of carcinoma *in situ (*CIS), lymphovascular invasion, and sessile tumor architecture (15, 16). It is noteworthy that the location of the tumor, such as renal pelvis, upper third ureter, middle third ureter, and lower third ureter, has not been described as a predictive factor of lymph node involvement (17). When present, these aggressive markers should be considered as an indication to perform LND.

With regards to cN+ patients, the indication of lymphadenectomy remains debated. First, CT-scan has a limited performance for the evaluation of lymph node involvement, defined as having at least a suspicious lymph node with more than 1 cm on preoperative imaging, with a sensitivity of 60% and a specificity of 82% (17). FDG-PET/CT could be better diagnose LN metastases in patients with UTUC with a sensitivity of 82% and a specificity of 84% (18). Then, most of the authors excluded cN+ patients from their studies, considering that pN+ patients have a systemic disease and would not benefit from RNU but from chemotherapy, extrapolating to cN+ patients. Among them, responders to the systemic treatment could benefit from surgery a second time after disease reevaluation. Some authors decided to perform LND when enlarged nodes were seen on preoperative imaging or when pathological nodes were discovered during surgery. In these cases, as pN+ is an indication of adjuvant chemotherapy, it is difficult to assign the survival gain to lymphadenectomy and not to the adjuvant treatment (19). Even though there is no consensus about the indication of lymph node dissection in UTUC management, factors associated with a high-risk of disease recurrences, such as cT2-4, high grade on biopsy, positive urine cytology, and large tumors, may indicate lymph node dissection (13–16). On the contrary, small, unifocal low-grade non-infiltrating tumors with negative cytology could be managed endoscopically. In these cases, LND is not required (1).

### Staging, Prognostication, and Decision Making of LND

As for disease stage, high-grade, lymphovascular invasion and concomitant CIS, lymph node involvement is a predictive factor of increased both cancer-specific and overall mortality (1, 9, 15). Table 1 lists patients' outcomes in the different studies analyzed in our review according to nodal status. Ikeda et al. (27) reviewed 404 patients with organ confined UTUC. The 5-year disease-free survival (DFS) and cancer-specific survival (CSS) rate were higher in pN0 patients than in pN+ patients, 84.5% vs. 43.6% (*p* < 0,001) and 78.3% vs. 33.2% (*p* < 0,001) respectively. These data are supported by those from Mason et al. (24). In their analysis, the 5-year recurrence-free survival (RFS) rate was 39% for pN0 patients and 7% for pN+ (HR = 2.94; 1.32–6.55). Similarly, the disease-specific survival (DSS) rates were 72.1 vs. 29.8% (HR = 2.9; 1.47–6.01) and overall survival (OS) rates were 66 vs. 22.3% (HR = 2.97; 1.47–6.01). In another study conducted among preoperatively node-negative patients, CSS and OS rates were higher in pN0 patients compared to those with pNx and pN1–3 disease, and these results were consistent across all tumor stages (33). Thus, pN+ patients' harbor decreased survival rates compared to pN0, highlighting the negative prognostic impact of lymph node involvement. As nodal involvement is an indication for adjuvant chemotherapy administration as demonstrated by the POUT trial (34), an accurate evaluation of the nodal status is needed for a proper selection of patients who may benefit from adjuvant systemic therapies.

**Table 1:** Staging role of LND.

**Study**	**Study design**	**Sample size**	**Nodal status**	**2-year DFS (%)**	**5-year DFS (%)**	**2-year CSS (%)**	**5-year CSS (%)**	**5-year OS (%)**	**Median follow-up (months)**	**Median number of LNs removed**	**Lymph node density**	**Surgical approach**	**Post-operative complications**	**Adjuvant or Neoadjuvant therapies**	**Reference**
Brown et al. (20)	Monocentric retrospective	184	pN0 48 % (89	NS	47.4	90	78	70.3	30 (0.1–17.9)	NS	NS	Open 86% (158); Laparosopic with open bladder-cuff excision 14% (26)	NS	NS	Nephroureterectomy for treating upper urinary tract transitional cell carcinoma: time to change the treatment paradigm?
			pNx 39 % (71)		47.4	88	78	70.3							
			pN+ 13% (24)		NS	52	37.6	33.9							
Kondo et al. (13)	Monocentric retrospective	181	pN0 77% (139)	NS	NS	95.2	85.2	NS	NS	6 (2–30)	3.7 (0 −16) (absolute number)	NS	No complication reported	NS	Primary Site and Incidence of Lymph Node Metastases in Urothelial Carcinoma of Upper Urinary Tract
			pNx/pN + 18% (32)/5 % (10)			26.3	15.5								
Brausi et al. (21)	Multicentric retrospective	82	pN0/pN + 29% (24)/20% (16)	64.3	NS	81.6	NS	80	64.7 (27 −288)	NS	NS	Open transperitoneal 90% (74); Open flank 10% (8)	NS	Adjuvant chemotherapy 4% (3); Adjuvant radiotherapy 1% (1)	Retroperitoneal Lymph Node Dissection in Conjunction with Nephroureterectomy in the Treatment of Infiltrative Transitional Cell Carcinoma of the Upper Urinary Tract: Impact on Survival
			pNx 51% (42)	46.3		44.8		30						0	
Secin et al. (17)	Monocentric retrospective	252	pN0 41% (105)	NS	NS	NS	56	NS	37	4 (2–10)	NS	Open 98%; Laparoscopic 2%	NS	Adjuvant chemotherapy 7% (17); Neoadjuvant chemotherapy 3% (7); Adjuvant or neoadjuvant unknown 3% (7)	Evaluation of regional lymph node dissection in patients with upper urinary tract urothelial cancer
			pNx 11% (28)				73								
			pN+ 48% (119)				0								
Roscigno et al. (22)	Monocentric retrospective	132	pN0 52% (69)	NS	72	NS	73	NS	42 (2–191)	8 (2–24)	NS	Open 100%	NS	Adjuvant chemotherapy 7.5% (10)	Prognostic Value of Lymph Node Dissection in Patients with Muscle-Invasive Transitional Cell Carcinoma of the Upper Urinary Tract
			pNx 20% (27)		39		48								
			pN+ 28% (36)		35		39								
Cho et al. (23)	Monocentric retrospective	152	pN0 35% (54)	NS	59.5	NS	72.3	NS	53 (6–214)	6 (1–35) (mean)	NS	Open 100%	NS	Adjuvant chemotherapy 31% (47)	Clinical Significance of Lymph Node Dissection in Patients with Muscle-Invasive Upper Urinary Tract Transitional Cell Carcinoma Treated with Nephroureterectomy
			pNx 58% (89)		58.2		62.7								
			pN+ 6% (9)		29.6		66.7								
Roscigno et al. (14).	Multicentric retrospective	1130	pN0 36 % (412)	NS	71	NS	77	NS	45 (1–250)	NS	NS	Open 82% (924); Laparoscopic 18% (206)	NS	Adjuvant chemotherapy 16.6% (187)	Impact of Lymph Node Dissection on Cancer Specific Survival in Patients With Upper Tract Urothelial Carcinoma Treated With Radical Nephroureterectomy
			pNx 51% (578)		66		69								
			pN+ 13% (140)		29		35								
Lughezzani et al. (10)	Multicentric retrospective	2842	pN0 64% (1835)	NS	NS	NS	81.2	NS	43 (1–203)	NS	NS	NS	NS	NS	A Critical Appraisal of the Value of Lymph Node Dissection at Nephroureterectomy for Upper Tract Urothelial Carcinoma
			pNx 26% (747)				77.8								
			pN+ 9% (242)				34.2								
Mason et al. (24)	Multicentric retrospective	1029	pN0 20% (199)	NS	90.9 (local DFS)	NS	72.1	NS	19.8 (7.2 −53.8)	4.3 (mean)	20% (mean)	Open 57% (583); Laparoscopic 43% (446)	NS	Adjuvant chemotherapy 10.9% (112)	The Contemporary Role of Lymph Node Dissection During Nephroureterectomy in the Management of Upper Urinary Tract Urothelial Carcinoma: The Canadian Experience
			pNx 73% (753)		70.6 (local DFS)		74.7								
			pN+ 7% (77)		80 (local DFS)		29.8								
Burger et al. (25)	Multicentric retrospective	785	pN0 17% (136)	NS	71.6	NS	79	NS	34 (15–65)	3 (2–6)	NS	Open 91% 715; Laparoscopic 9% (70)	NS	Adjuvant chemotherapy 9% (69)	No overt influence of lymphadenectomy on cancer-speciWc survival in organ-conWned vs. locally advanced upper urinary tract urothelial carcinoma undergoing radical nephroureterectomy: a retrospective international, multi-institutional study
			pNx 76% (595)		76.9		77.4								
			pN+ 7% (54)		21.3		26.7								
Yoo et al. (26)	Monocentric retrospective	418	pN0 29% (116)	NS	76.4	NS	NS	80.2	69	7 (3–10)	NS	Open 37% (106); Minimal invasive* 63% (180)	NS	NS	Does lymph node dissection during nephroureterectomy affect oncological outcomes in upper tract urothelial carcinoma patients without suspicious lymph node metastasis on preoperative imaging studies?
			pNx 68% (286)		73.4			71.7							
			pN+ 3% (16)		93.7			12.5							
Ikeda et al. (27)	Multicentric retrospective	404	pN0 45% (182)	NS	78.3	NS	84.5	NS	43 (17–89)	6 (3–10)	NS	Open 74% (296); Laparoscopic 26% (103)	NS	Adjuvant chemotherapy 19% (74)	Effect of Lymphadenectomy During Radical Nephroureterectomy in Locally Advanced Upper Tract Urothelial Carcinoma
			pNx 45% (177)		61.9		73.3								
			pN+ 10% (40)		33.2		43.6								
Inokuchi et al. (28)	Multicentric retrospective	2037	pN0 47% (955)	NS	NS	NS	NS	69.3	45.8 (21.8 −75.9)	6 (3–11)	NS	Open 60.5% (1234); Laparoscopic 38.6% (787)	NS	Adjuvant chemotherapy 5%; Neoadjuvant chemotherapy 3% (71)	Role of lymph node dissection during radical nephroureterectomy for upper urinary tract urothelial cancer: multi-institutional large retrospective study JCOG1110A
			pNx 42% (859)					60.5							
			pN+ 11% (223)					30							
Lenis et al. (29)	Multicentric retrospective	3116	pN0 83% (2594)	NS	NS	NS	NS	NS	NS	3 (1–7)	NS	Open 32% (969); Laparoscopic 44% (1385); Robotic 24% (762)	NS	Adjuvant chemotherapy 12.8% (400); Neoadjuvant chemotherapy 1.9% (60)	Role of surgical approach on lymph node dissection yield and survival in patients with upper tract urothelial carcinoma
			pNx 12% (60)												
			pN+ 6% (162)												
Dong et al. (30)	Multicentric retrospective	2731	pN0 18% (491	NS	NS	NS	NS	54	31	2 (1–5)	NS	NS	NS	Adjuvant chemotherapy 12.6% (345); Adjuvant radiotherapy 3.3% (90)	Lymph node dissection could bring survival benefits to patients diagnosed with clinically node-negative upper urinary tract urothelial cancer: a population-based, propensity score-matched study
			pNx 82% (2240)					47							
			NS					NS							
Sato et al. (31)	Monocentric retrospective	68	pN0 85% (58	NS	NS	NS	NS	85	49.5 (3–140)	12 (3–34)	NS	NS	NS	Adjuvant chemotherapy 32.4% (22)	Prognostic assessments in patients with upper tract urothelial carcinoma undergoing radical nephroureterectomy and systematic regional lymph node dissection
			pNx NS					NS							
			pN+ 15% (10)					55							
Li et al. (32)	Multicentric retrospective	1340	pN0 21% (278)	NS	NS	NS	NS	NS	NS	NS	NS	Hand-assisted 55% (741); Pure laparoscopic 34% (458); Robotic 11% (141)	Clavien-Dindo > 2: 6% (80)	Adjuvant chemotherapy 23% (311); Adjuvant immunotherapy 0.8% (11)	Comparing Oncological Outcomes and Surgical Complications of Hand- Assisted, Laparoscopic and Robotic Nephroureterectomy for Upper Tract Urothelial Carcinoma
			pNx 75% (1004)												
			pN+ 4% (58)												

*NS, Not Specified*.

### Therapeutic Role of LND (Survival Benefit)

Beyond its prognostic utility, LND plays a potential therapeutic role in survival of locally advanced tumors (35). However, LND is not without consequences and may lead to longer operative time and increased postoperative complications. The indication of LND and its extent must be discussed as the impact of lymphadenectomy itself on survival is to be proven. Zhai et al. studied the effect of LND during RNU on cancer-specific survival and overall survival (33). They retrospectively analyzed 7,278 patients from SEER database with histologically proven UTUC, who underwent RNU with or without LND. All patients were node-negative preoperatively. In multivariable analyses, patients who underwent LND had a decreased CSS (HR 0.81; *p* < 0.01) compared to patients operated without LND, with a 5-year CSS rate of 65.8 vs. 74.3%. These results were consistent in OS (HR 0.87; *p* < 0.01), with a 5-year OS rates of 41.5 vs. 47.1%. After the analyses were repeated across all pathological stages, the benefice on OS of LND remained consistent for patients with pT3-4 disease, but not for pT1-2 patients. Moreover, patients receiving extended LND namely > 3 LNs removed or limited LND namely 1–3 LNs removed had higher OS rate compared to no LND with respective HR of 0.83 (*p* < 0.01) and 0.90 (*p* < 0.05). When stratifying according to tumor stage, the beneficial impact of extended LND on OS was only found in pT3 (HR 0.80, *p* < 0.05) or pT4 stages (HR 0.77, *p* < 0.05). On the contrary, Roscigno et al. (14) showed that there were no statistically significant differences between patients who underwent LND and those who did not. In their retrospective multicenter study, the 5-year DFS rate was 60% when LND was performed vs. 65% for no LND patients (*p* = 0.12), and the 5-year CSS rate was 66% for LND patients vs. 69% for no LND group (*p* = 0.23). It is to be noted that both DFS and CSS increased incrementally from pN+ to pNx to pN0 patients when LND was performed compared to no LND. This suggests that pN0 cases might beneficiate from lymphonodal excision in having possible micrometastasis removed, and pN+ patients from systemic adjuvant chemotherapy administration before surgery. However, half of the patients had an unknown nodal status (pNx), which alters the validity of the conclusions. The benefit of lymphadenectomy on survival is therefore still under debate, likewise for its anatomical template and extent. Yet, studies tend to show prolonged survival when LND is performed (Table 2). The latter are mostly biased by their retrospective design and low recruitment. Stronger evidence is awaited in this field.

**Table 2:** Therapeutic role of LND.

**Study**	**DFS: pN0 vs pN+**	**DFS: pN0 vs pNx**	**DFS: pN+ vs pNx**	**CSS: pN0 vs pN+**	**CSS: pN0 vs pNx**	**CSS: pN+ vs pNx**	**OS: pN0 vs pN+**	**OS: pN0 vs pNx**	**OS: pN+ vs pNx**
Brown et al. (20)	NS	*p* = 0.58	NS	NS	*p* = 0.85	NS	NS	NS	NS
Kondo et al. (13, 36)	NS	NS	NS	NS	NS	NS	NS	NS	NS
Brausi et al. (21)	NS	NS	NS	NS	NS	NS	NS	NS	NS
Secin et al. (17)	NS	NS	NS	HR 3.38 (1.82–6.25)	0.81 (0.48–1.36)	NS	NS	NS	NS
Roscigno et al. (22)	*p* < 0.001	*p* < 0.001	*p* < 0.001	*p* < 0.001	*p* < 0.001	*p* = 0.476	NS	NS	NS
Cho et al. (23)	HR 2.45 (0.27–22.5)	HR 3.91 (1.35–11.33)	NS	*p* > 0.05	*p* > 0.05	*p* > 0.05	NS	NS	NS
Roscigno et al. (14)	HR 2.185 *p* < 0.001	HR 1.4 (*p* = 0.018)	*p* < 0.001	HR 2.12 *p* < 0.001	HR 1.42 (*p* = 0.016)	*p* = 0.024	NS	NS	NS
Lughezzani et al. (10)	NS	NS	NS	HR 2.54 (*p* < 0.001)	HR 0.99 (*P* = 0.9)	NS	NS	NS	NS
Mason et al. (24)	HR 2.94 (1.32–6.55)	HR 1.23 (0.78–1.96)	HR 2.83 (1.54–5.18)	HR 2.97 (1.47–6.01)	HR 0.96 (0.64–1.44)	HR 2.70 (1.56–4.69)	HR 2.97 (1.47–6.01)	HR 0.96 (0.64–1.44)	HR 2.70 (1.56–4.69)
Burger et al. (25)	*p* < 0.001	*p* = 0.586	*p* < 0.001	*p* < 0.001	*p* = 0.985	*p* < 0.001	NS	NS	NS
Yoo et al. (26)	NS	*p* = 0.682	NS	NS	NS		NS	*p* = 0.230	NS
Ikeda et al. (27)	*p* < 0.001	*p* < 0.001	NS	*p* < 0.001	*p* < 0.001	NS	NS	NS	NS
Inokuchi et al. (28)	NS	NS	NS	HR 1.91 (*p* = 0.003)	NS	NS	HR 5.67 (4.56–7.05)	HR 1.03 (0.83–1.27)	NS
Lenis et al. (29)	NS	NS	NS	NS	NS	NS	HR 1.87 (1.47–2.37)	HR 1.03 (0.85–1.25)	NS
Dong et al. (30)	NS	NS	NS	NS	HR 0.779 (0.661–0.918)	NS	NS	0.788 (0.644–0.965)	NS
Sato et al. (31)	NS	NS	NS	HR 1.38 (*p* = 0.47)	NS	NS	HR 4.57 (*p* = 0.021)	NS	NS
Li et al. (32)	NS	NS	NS	HR 4.405 (2.557, 7.589)	HR 1.141 (0.779, 1.670)	NS	HR 3.079 (1.959, 4.838)	HR 1.097 (0.836, 1.440)	NS

*NS, Not Specified*.

### How to Do LND

#### Anatomical Template

**Figure 1:**
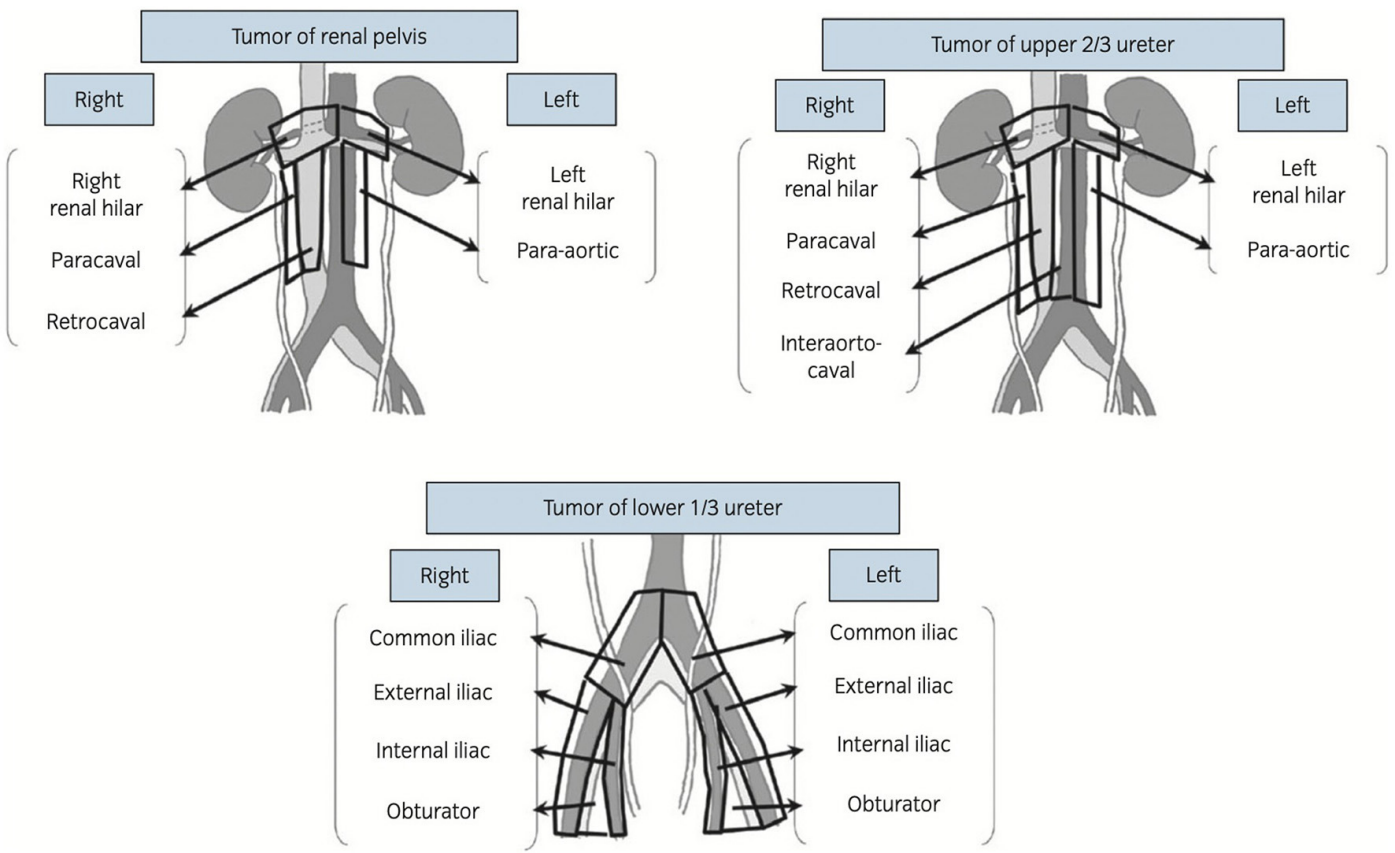
Regional lymphovascular drainage depending on the location of the primary tumors [according to Kondo et al. (37)].

Although there are nor specific guidelines either from the European Association of Urology or from the American Urological Association concerning the anatomical template of lymph node excision, studies tend to agree on the anatomical regions concerned by lymph node invasion in UTUC (38). The latter are represented by the renal hilar, para-aortic, inter-aorto-caval, retro-caval, latero-caval, and pelvic areas. The upper urinary tract is therefore divided into eight parts based on its lymphovascular drainage: the left renal pelvis, the left upper ureter, the left middle ureter, the left lower ureter, the right renal pelvis, the right upper ureter, the right middle ureter, and the right lower ureter. The upper ureter is defined as the upper third of the ureter, above the inferior mesenteric artery. The middle ureter goes from the inferior mesenteric artery to the common iliac artery. Finally, the lower ureter is the distal third of the ureter from the crossing of the common iliac artery to the ureteral meatus (Figure 1).

Kondo et al. retrospectively reviewed the primary site and incidence of nodal metastases in UTUC (13). They based their analyses on the imaging and pathological exam of surgical specimens. Of 181 patients treated with RNU, regional lymph node invasion was found in 42 (23%) of them. Of these 42 patients, only 10% had nodal involvement on preoperative imaging. The incidence of nodal involvement was more frequent for tumors of the renal pelvis, upper ureter, and middle ureter than the lower ureter (20–30 vs. 10%). Concerning tumors of the right renal pelvic, the primary sites of nodal involvement were the right hilar, the paracaval, and the retrocaval areas whereas for tumors of the left renal pelvic they were the left hilar, abdominal paraaortic, and interaorto-caval nodes. It is to be noted that there were no lymph node metastases above the renal hilus. In the tumors of the right upper ureter, metastases were found at the retrocaval node and interaortocaval node. Tumors of the right middle ureter metastasized to the interaortocaval and retrocaval nodes. Nodal metastases of the right lower ureter tumor were observed at the common iliac node and obturator node. There was no nodal metastasis in tumors of the left upper ureter, but metastasis of the tumors of the left middle ureter was observed at the abdominal para-aortic nodes. Finally, the left lower ureter tumor metastasized at the common iliac node and internal iliac node.

These findings highlighted several points:

- The primary site of lymph node metastasis depends on the primary tumor location.
- It seems there is no crossing over concerning lymphovascular drainage when the tumor is located above the common iliac artery.
- No nodal metastasis is observed above the aortic bifurcation when the primary tumor is located on the lower ureter.
- Right renal pelvic, right upper, and middle ureter tumors metastasis to homolateral renal hilar, para-caval, retro-caval, and inter-aorto-caval nodes.
- Left renal pelvic, left upper, and middle ureter tumors metastasis to homolateral renal hilar, para-aortic nodes.
- Tumors of the lower ureters metastasis to pelvic nodes, including common iliac, external iliac, internal iliac, and obturator nodes.

Since then, several studies confirmed these principles and anatomical templates of LND with a benefit from an oncological point of view (14, 36, 39). Nevertheless, these findings must be tempered by the retrospective design and the low number of tumors studied at each location. Prospective studies are evaluating the appropriate template of LND in cN0 patients on oncologic outcomes and safety (40, 41). Long terms results and controlled trials are needed to answer the question.

#### Quality Control (Number of Lymph Nodes to Remove and Lymph Node Density)

Despite the prognostic value and potential therapeutic effect of lymph node excision in UTUC, the benefit of an extended lymphadenectomy during RNU remains debatable (42). It is well-known that the number of LNs removed affects survival after radical cystectomy in both negative- and positive-node patients (43). Parallelism could be made between upper tract urothelial cancer and muscle-invasive bladder cancer. Although anatomic studies well-describe the variability of pelvic lymph nodes in patients undergoing lymphadenectomy during cystectomy, there is a lack of information concerning lymph nodes distribution in LND templates during RNU (44).

Roscigno et al. (12) published an international multicenter retrospective cohort of 552 patients treated by RNU with lymphadenectomy for non-metastatic UTUC. Patients with pTa-Tis disease were excluded owing to the negligible risk of positive lymph nodes. The extent of the lymph node excision was at the surgeon's discretion based on clinical presentation, location, and laterality of the primary tumor. Lymphadenectomy usually included para-aortic, latero-caval, and inter-aorto-caval nodes from the renal pelvic to the inferior mesenteric artery in case of tumors of the renal pelvic and upper third ureter. In the case of mid ureteral cancer, nodes from the renal hilum to the bifurcation of the common iliac artery were removed. Finally, in the case of lower ureteral tumors, the anatomical template of lymphadenectomy concerned the ipsilateral pelvic nodes. The median number of LNs removed was four (range: 1–38) for patients with UTUC of the renal pelvis and five (range: 1–41) for UTUC of the ureter. In this study, the number of LNs removed in the whole population was independently associated with recurrence (HR: 0.97; *p* = 0.04), but not with cancer-specific mortality (CSM) (*p* = 0.1). Surprisingly, when analyzing by subgroup, in the case of pN0, the number of LNs removed was associated with recurrence (HR: 0.97; *p* = 0.03) and with cancer-specific mortality (HR: 0.96; *p* = 0.04), whereas in the case of pN+, the number of LNs removed was neither associated with recurrence (*p* = 0.48) nor with cancer-specific mortality (*p* = 0.74). In pN0 patients, the CSM decreases as the number of LNs removed increases. The most informative cut-off for the number of LNs removed was eight, for recurrence as well for CSM. In multivariable analyses, the dichotomized variable of eight LNs removed predicted independent recurrence (HR: 0.49; *p* = 0.005) and CSM (HR: 0.40; *p* = 0.003). This shows that extended lymph node dissection results in lower recurrence rates and increased cancer-specific survival in pN0 UTUC patients treated by RNU. The extent of lymphadenectomy should improve disease staging by better stratifying node involvement. An extended lymphadenectomy may provide therapeutic advantages in pN0 patients with UTUC as a higher number of nodes removed in pN0 patients may be related to the removal of undetected LN micrometastases and the subsequent improvement in survival. However, these results contrast with the retrospective study from Winer et al. evaluating the extent of LND on oncologic outcomes and safety (45). In the whole population, they found no association between the extent of LND and RFS or CSS. When stratifying on nodal status, it appeared that pN+ patients had an improved RFS (*p* = 0.039) but no improvement in CSS (*p* = 0,6). Regarding pN0 patients, there was no evidence of an association between nodal yield and RFS (*p* = 0.8) or CSS (*p* = 0.6).

Despite the evidence of the potential prognostic and therapeutic roles of extended LND for UTUC, many patients undergoing RNU have the number of removed nodes less than eight (46). As a matter of fact, some authors consider that the number of LNs removed is not a relevant indicator to evaluate the effect of LND on patient's outcomes, introducing LN density. It is defined as the percentage of the number of LNs involved with tumor divided by the total number of LNs examined. Althoughit has been evaluated in bladder cancer, its place in the management of UTUC is uncertain (47). Bolenz et al. (48) evaluated the effect of LND on recurrence-free and disease-free survival in a retrospective cohort of patients undergoing RNU with regional lymphadenectomy. Of the 432 patients, 135 (31%) had LN metastases. The median number of LNs removed was four. In multivariable analyses, when lymph node density was superior to 30%, the risk of cancer recurrence (25 vs. 38%; HR: 1.8; *p* = 0.021) and cancer mortality (30 vs. 48%; HR: 1.7; *p* = 0.032) were significantly higher. Mason et al. (24) confirmed that the ratio of positive nodes to the total number of LNs removed was associated with decreased recurrence-free survival (HR: 1.94; *p* = 0.015) and overall survival (HR: 2.34; *p* = 0.0013) with a cut-off of > 20%. Lymph node density seems to be an interesting marker to predict outcome after LND, whereas the number of LNs is inferior or superior to eight.

#### Surgical Approach and Safety

Radical nephroureterectomy can be performed with open techniques as well as laparoscopically +/- robot-assisted with similar oncological and safety outcomes (49, 50). The time of lymph node excision can independently be performed laparoscopically or through an open technique. Pearce et al. (51) examined the effect of surgical approach on regional lymphadenectomy (LND) performance and safety for radical nephroureterectomy. A total of 16,619 patients were prospectively included for analysis. Patients undergoing robotic NU were more likely to undergo LND (27%) when compared to the open-surgical approach (15%) and laparoscopic approach (10%) (*p* < 0.001). Peyronnet et al. (50) reviewed the percentage of LND comparing open technique to a laparoscopic procedure. Of the 22 studies in which lymph node dissection indication was reported, only three reported significantly lower rates of lymphadenectomy in the laparoscopic group compared to the open approach. Regarding the number of lymph nodes removed, only one study reported a significant difference favoring the open over the laparoscopic approach. These results are to be balanced with those from Roscigno et al. (12) and Abe et al. (52) who reported a comparable median LN number between the open group and the pure laparoscopic cohort. Finally, two studies based on the US National Cancer Database found that a higher proportion of LND was performed when patients were operated by open or robot-assisted techniques than by pure laparoscopic approach (29, 53). These contradictory results are explained by the many biases emanating from the retrospective design and the center effect of the studies analyzed.

Regarding oncological results, it appears that there is no difference concerning the surgical technique. Comparable results can be drawn on cancer recurrence and mortality between open and laparoscopic groups (14). Abe et al. (52) described similar 5-year RFS, 5-year CSS, and 5-year OS between patients treated with open and laparoscopic procedures. These results are consistent with those from Kido et al. (54) in their high-volume monocenter study. However, we must put these results into perspectives as laparoscopic RNU is generally performed in patients with more favorable pathologic features conducting to selection bias.

Concerning safety, only one prospective study was designed to assess LND complications (19). A total of 19 patients were prospectively included with a mean number of LNs removed of 7 and a median follow-up of 12 months. Of these, eight had minor complications (Clavien Grade I–II) and only one patient had a major complication, which consisted of a postoperative lymphatic leak requiring a second surgical procedure (Clavien grade IIIb). In the study from Pearce et al., multivariate analysis revealed that patients undergoing LND were 30% more likely to experience any postoperative complication (51). As for the surgical approach, there was no significant difference in the rate of intraoperative complications. The rate of postoperative complication was lowest for robot-assisted NU and highest for open NU (*p* < 0.001) with significant differences in the rate of gastrointestinal and hemorrhagic complications.

## Conclusion

Since the first description of RNU in 1978, the knowledge of lymph node dissection has evolved. The negative prognostic impact of lymph node involvement has been proven, indicating LND to at least improve staging and help selecting patients for systemic adjuvant chemotherapy. The therapeutic role of lymph node dissection remains to be demonstrated but most of studies tend to show a better survival when LND is realized, especially in locally advanced tumors. The anatomical template of lymph node excision and the number of lymph nodes to be removed are still discussed, but a predefined anatomical pattern adapted to the location of the primary tumor, and a minimum of 8 nodes removed seem to enhance the disease management and survival. Concerning the surgical approach, there is no significant difference in performance and safety between open, laparoscopic, and robotic-assisted RNU. Well-designed prospective controlled studies are needed to better indicate lymph node dissection in high-risk non-metastatic upper tract urothelial cancers.

## Author Contributions

All authors listed have made a substantial, direct, and intellectual contribution to the work and approved it for publication.

## Conflict of Interest

The authors declare that the research was conducted in the absence of any commercial or financial relationships that could be construed as a potential conflict of interest.

## Publisher's Note

All claims expressed in this article are solely those of the authors and do not necessarily represent those of their affiliated organizations, or those of the publisher, the editors and the reviewers. Any product that may be evaluated in this article, or claim that may be made by its manufacturer, is not guaranteed or endorsed by the publisher.
